# Eclipsed Acetaldehyde as a Precursor for Producing Vinyl Alcohol

**DOI:** 10.3390/ijms131115360

**Published:** 2012-11-20

**Authors:** Osman I. Osman, Abdulrahman O. Alyoubi, Shabaan A. K. Elroby, Rifaat H. Hilal, Saadullah G. Aziz

**Affiliations:** Chemistry Department, Faculty of Science, King Abdulaziz University, P.O. Box 80203, Jeddah 21589, Saudi Arabia; E-Mails: aalyoubi@kau.edu.sa (A.O.A.); Skamel@kau.edu.sa (S.A.K.E.); rhilal@kau.edu.sa (R.H.H.); saziz@kau.edu.sa (S.G.A.)

**Keywords:** acetaldehyde, eclipsed, bisected, vinyl alcohol, tautomerization, hyperconjugation, MP2, B3LYP, NBO

## Abstract

The MP2 and DFT/B3LYP methods at 6-311++G(d,p) and aug-cc-pdz basis sets have been used to probe the origin of relative stability preference for eclipsed acetaldehyde over its bisected counterpart. A relative energy stability range of 1.02 to 1.20 kcal/mol, in favor of the eclipsed conformer, was found and discussed. An NBO study at these chemistry levels complemented these findings and assigned the eclipsed acetaldehyde preference mainly to the vicinal antiperiplanar hyperconjugative interactions. The tautomeric interconversion between the more stable eclipsed acetaldehyde and vinyl alcohol has been achieved through a four-membered ring transition state (TS). The obtained barrier heights and relative stabilities of eclipsed acetaldehyde and the two conformers of vinyl alchol at these model chemistries have been estimated and discussed.

## 1. Introduction

Acetaldehyde (CH_3_CHO) was first synthesized by Scheele in 1774 [1]. The importance of acetaldehyde lies in its usage as an intermediate in many organic reactions [2]. To understand the mechanisms of how acetaldehyde works as an intermediate in these organic reactions; the relative stability of its different conformers resulting from the internal rotation of the C–O bond should be investigated. The internal rotation of C–O bond in acetaldehyde produces two stable conformers; namely the eclipsed and bisected forms. Kilb *et al.*[3] reported an experimental internal rotation barrier of 1.162 kcal/mol in favor of eclipsed conformer over that of a bisected one. Souter and Wood [4] using far-infrared data determined a new value of 1.128 kcal/mol. A SPASIBA force filed calculations [5] yielded a rotation barrier of 1.22 kcal/mol. Using MP2/6-311G(d,p), Munoz-Caro *et al.*[6] calculated a torsional energy of 1.164 kcal/mol. There seems to be poor agreement between the observed and calculated values. We are attempting in this paper, mainly to investigate the origin of the preference of the eclipsed form, and hopefully settle the slight conflict between experiment and theory. The thermolysis of acetaldehyde at temperatures from 480 °C to 525 °C broke the weakest C–C σ bond that needed an activation energy of 47.6 kcal/mol [7] that produced C_2_H_6_ as a terminating step. The NMR studies of the photolysis of acetaldehyde confirmed the presence of vinyl alcohol [8]. Vasiliou *et al.*[9,10] investigated the production of vinyl alcohol by the pyrolytic abstraction of the aldehyde hydrogen followed by migration of one the methyl hydrogens to the other carbon atom.

Holmes and Lossing [11] estimated the relative energy of acetaldehyde to vinyl alcohol of 41 ± 8 kJ/mol in favor of the former. Smith *et al.*[12] computed an activation energy of 67.38 kcal/mol for the lower energy route of converting symmetrical acetaldehyde to vinyl alcohol. Their suggestion is in conflict with other routes deduced from experiment [13,14]. Andres *et al.*[15] using MP2/6-311G ** level of theory, estimated an activation energy of 72.45 kcal/mol for the conversion of symmetrical acetaldehyde into vinyl alcohol; and a relative energy of 11.97 kcal/mol in favor of the former. In the second part of paper, we endeavor to reexamine the mechanism of the tautomerization reaction of the more stable eclipsed acetaldehyde and vinyl alcohol, mainly through Natural Bond Orbital (NBO) calculations.

## 2. Results and Discussion

### 2.1. Relative Stability of Eclipsed and Bisected Acetaldehydes

#### 2.1.1. Relative Energy

**Figure 1:**
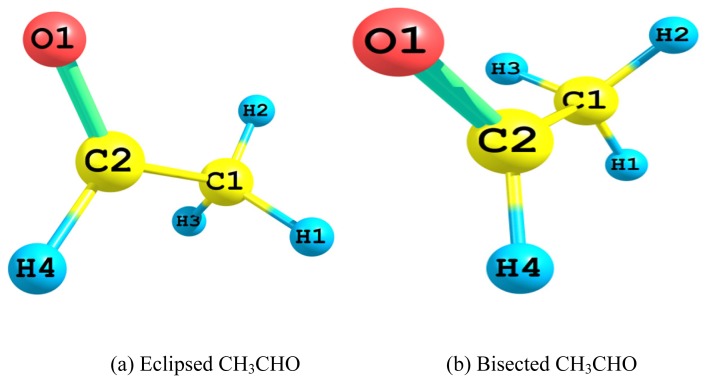
The atom type and labeling of Eclipsed and Bisected Acetaldehyde. (a) Eclipsed CH_3_CHO (b) Bisected CH_3_CHO

The atom type and labeling of the eclipsed (Figure 1a) and bisected (Figure 1b) conformers of acetaldehyde are depicted. In Table 1 are shown the two conformers total full electronic energies (*E*_Full_), total Lewis electronic energies (*E*_L_) and dipole moments, which were computed by using MP2 and B3LYP methods at 6-311++G(d,p) and aug-cc-pvdz basis sets. *E*_L_ is the total electronic energy resulting from the localized “natural Lewis structure” wave functions in which the orbitals are doubly occupied *i.e.*, it is the energy obtained by deleting all non-Lewis orbitals from the basis sets. It is useful in studying hyperconjugative interactions that are used in the quantitative study of relative stabilities and their origins. *E*_Full_ is the total electronic energy resulting from the original delocalized wave functions. The energy differences (Δ*E*_T_) between their full electronic energies (1.02–1.21 kcal/mol) using these model chemistries are in fair agreement with that of 1.162 kcal/mol obtained by experiment [3] in favor of the eclipsed conformer. It is apparent that these energy differences are both chemistry model and basis set dependent. In comparison to the observed [3] value; the aug-cc-pvdz basis set estimated energy differences that deviated by 2.40% and 4.13%; whereas the 6-311++G(d,p) basis set gave larger deviations of *ca.* 12.22% and 6.20%; at the MP2 and B3LYP theoretical methods, respectively. This non-satisfactory agreement between the experimental value [3] of 1.162 kcal/mol and our calculated values sheds some doubts about the former as it is also in conflict with other experimental values [4–6].

#### 2.1.2. Hyperconjugative Interactions

The Natural Bond Bond (NBO) analysis of the calculated Lewis energies for the two conformers using 6-311++G(d,p) and aug-cc-pvdz basis sets at B3LYP method gave energy differences of 4.59 and 5.15 kcal/mol, respectively, in favor of the bisected conformer; and delocalization energies of 5.68 and 6.35 kcal/mol, respectively, in favor of the eclipsed one. Thus, these basis sets gave total energy gains (Δ*E*_T_) of 1.09 and 1.20 kcal/mol, respectively, with a preference for the eclipsed conformer. The relative stabilization energies, therefore, deviated by 6.20% [6-311++G(d,p)] and 3.27% [aug-cc-pvdz] from the observed value [3] of 1.162 kcal/mol. The NBO energy analysis gave the same results as those obtained by the analysis of *E*_Full_. It was thus not helpful in resolving the conflict; but our main objective, in using NBO analysis, was to answer the fundamental question: what is the origin of the competiveness of the eclipsed conformer?

The Lewis structure preference for the bisected conformer can safely be explained in terms of the relatively minimal steric hindrance. To analyze the delocalization energy preference for the eclipsed counterpart; Natural Bond Order (NBO) theory [17,18] has proved to be extremely helpful in determining the quantities and origins of the relative stabilities of conformers [19]. The delocalization energy lowering caused by hyperconjugative energies can safely be treated by the second-order perturbation energy (*E*_(2)_) given in the NBO theory as: (*E*_(2)_ = Δ*E_ij_* = *q_i_* (*F_ij_*)^2^/Δɛ) where *q*_i_ is the donor orbital occupancy, *F*_ij_ is the off-diagonal Kohn-Sham matrix elements between the occupied *i* (*n* or σ) and empty *j* (σ*) orbitals and Δɛ is the difference between the energy of the donor orbital (*i*) and the acceptor orbital (*j*).

In Table 2 are listed the most important second-order perturbation energy estimates of the hyperconjugative interactions of the eclipsed and bisected conformers of acetaldehyde using B3LYP/aug-cc-pvdz level. The two lone pairs (LP) on the oxygen atom interact with the C–C bond (n_O1_→σ*_CC_ and n_O2_→σ*_CC_) and C2-H4 bond (n_O1_→σ*_C2–H4_ and n_O2_→σ*_C2–H4_). These interactions gave total delocalization energies of 45.09 and 44.36 kcal/mol for the eclipsed and bisected conformers, respectively. Thus the LP Effect [20] favored the eclipsed form by 0.73 kcal/mol. The total energies of the vicinal CH–CO* and CH–CH* interactions are 20.26 and 17.56 kcal/mol for the eclipsed and bisected conformers, respectively. They favored the eclipsed form by 2.7 kcal/mol. The eclipsed conformer has two strong antiperiplanar (σ_C1–H2_→σ*_C2–H4_ and σ_C2–H4_→σ*_C1–H2_) interactions of 3.03 and 2.53 kcal/mol, which are absent in the bisected counterpart; but replaced by the antiperiplanar (σ_C1–H1_→σ*_CO_) interaction of 4.51 kcal/mol. The eclipsed form has two dual strong semi antiperiplanar (σ_C1–H1_→σ*_CO_ and σ_C1–H3_→σ*_CO_) interactions of a total of 7.35 kcal/mol each; whereas the bisected counterpart has four matching relatively weaker semi antiperiplanar (σ_C1–H2_→σ*_CO_, σ_C1–H3_→σ*_CO_, σ_C1–H2_→σ*_C2–H4_, σ_C1–H3_→σ*_C2–H4_) interactions of 4.68, 4.69, 1.34 and 1.34 kcal/mol. Thus due to these interactions the bisected conformer lies 2.7 kcal/mol above the eclipsed one. This outcome can be explained in terms of the C1–H2 and C2–H4 anti arrangement. This kind of geometrical environment allows greater overlap between the interacting orbitals [20]. In contrast, the bisected form C1–H2 and C2–O1 bonds have less favorable overlap between their interacting orbitals. Globally, the eclipsed conformer is stabilized by *ca.* 3.44 kcal/mol. About 78.7% of this energy gain stems from the vicinal periplanar interactions.

### 2.2. Tautomerization Reaction of Eclipsed Acetaldehyde and Vinyl Alcohol

#### 2.2.1. Geometry

**Figure 2:**
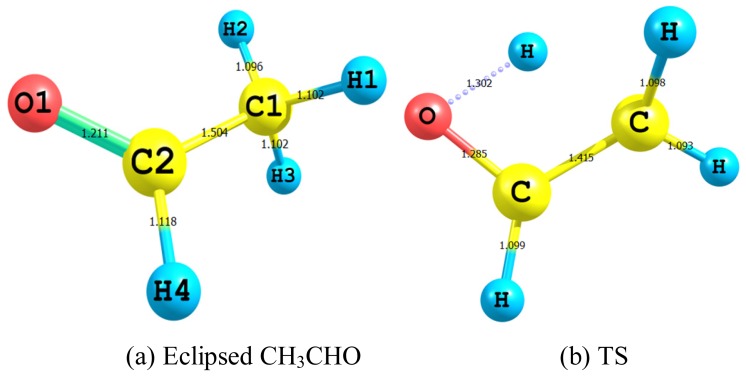
The atom type and labeling and the optimized bond lengths (Å) of eclipsed acetaldehyde and its TS calculated by using B3LYP/aug-cc-pvdz level of theory.

The shapes of eclipsed acetaldehyde (Figure 2a) and its transition state (TS) (Figure 2b); together with the optimized bond lengths (Å), which were calculated by using B3LYP/aug-cc-pvdz level of theory, are shown. Table 3 depicts the optimized geometries of eclipsed acetaldehyde, TS and syn and anti vinyl alcohol which were estimated by applying the same model chemistry. Apart from H1C1H3 and H4C2O angles which deviate by 1.27° and 2.47° respectively, our calculated bond lengths and angles of acetaldehyde are in satisfactory agreement with the experimental values [3,21]. For syn and anti vinyl alcohol, the average deviation between the observed [22,23] and computed parameters is 0.007 Å for the bond lengths and 0.56° for the bond angles.

The main geometrical features of the TS that manifested the interconversion between eclipsed acetaldehyde and vinyl alcohol are: (i) the elongation of the C1–H2 from 1.096 Å in acetaldehyde to become 1.507 Å in the TS and to disappear completely in vinyl alcohol (ii) the shortening of C–C bond of 1.504 Å in acetaldehyde to become 1.415 Å in the TS and to finally settling at 1.337 Å in vinyl alcohol as a C=C bond (iii) the elongation of acetaldehyde C=O bond of 1.212 Å by about 0.073 Å in the TS and to attain a typical length of a C–O bond length of 1.366 Å in vinyl alcohol (iv) the emergence of a weakly bonded (H–O) bond of 1.302 Å in the TS that eventually reached a normal HO bond length of 0.967 Å in vinyl alcohol (v) the typical sp^3^ HCH angles of *ca*. 109.6° in acetaldehyde opened up by about 4.2° in the TS and flattened up more by *ca*. 4.1° in vinyl alcohol (vi) the three-dimensional dihedral angles of acetaldehyde have worked out their path nicely toward a flat vinyl alcohol through the TS.

#### 2.2.2. Activation Energies and Relative Stabilities

**Figure 3:**
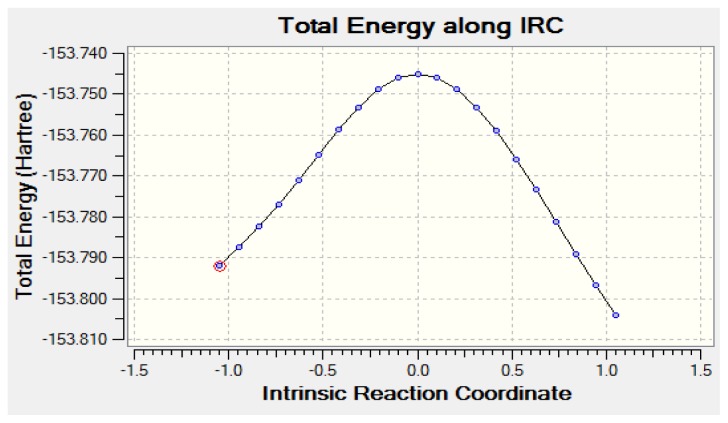
The potential energy profile of the thermal dissociation acetaldehyde along the IRC pathway calculated using B3LYP/aug-cc-pvdz level of theory.

Table 4 lists the zero-point corrected total electronic energies and activation energies of eclipsed acetaldehyde, the Transition State (TS) and syn and anti vinyl alcohol which were computed by using MP2 and B3LYP methods at 6-311++G(d,p) and aug-cc-pvdz basis sets. Figure 3 depicts the Intrinsic Reaction Coordinate (IRC) plot that connects eclipsed acetaldehyde to vinyl alcohol through the TS by using B3LYP/aug-cc-pvdz model chemistry. The analysis of the TS imaginary frequency showed variations in the C1–H2 bond length that were in line with the anticipated interconversion between the eclipsed acetaldehyde and vinyl alcohol which were connected by a saddle point.

The analysis of the results depicted in Table 4 shows that the activation energies and relative stabilities of eclipsed acetaldehyde and vinyl alcohol (Δ*E*_1_), in one hand, and between the two isomers of vinyl alcohol (Δ*E*_2_), on the other hand; are dependent on the levels of calculations. However, they are not far from each other and the trends are quite consistent. The B3LYP method predicted lower barrier heights (66.35 and 64.61 kcal/mol) compared to those estimated by MP2 model chemistry (67.47and 65.70 kcal/mol) using 6-311++G(d,p) and aug-cc-pvdz basis sets respectively. On the one hand, our calculated activation energy of 67.47 kcal/mol using the MP2/6-311++G(d,p) level deviated by *ca.* 5 kcal/mol compared to that predicted by Andres *et al.*[15] at the same level of calculation, but with uncorrected zero-point energies. However, it deviated by 0.006 kcal/mol when compared to that computed by Smith *et al.*[12] using G1 level of theory. On the other hand, our calculated relative stability of eclipsed acetaldehyde/vinyl alcohol of 12.85 kcal/mol exceeded that estimated by Andreas *et al.*[15] by 0.88 kcal/mol and by 1.6 kcal/mol when compared to that computed by Smith *et al.*[12].

**Figure 4:**
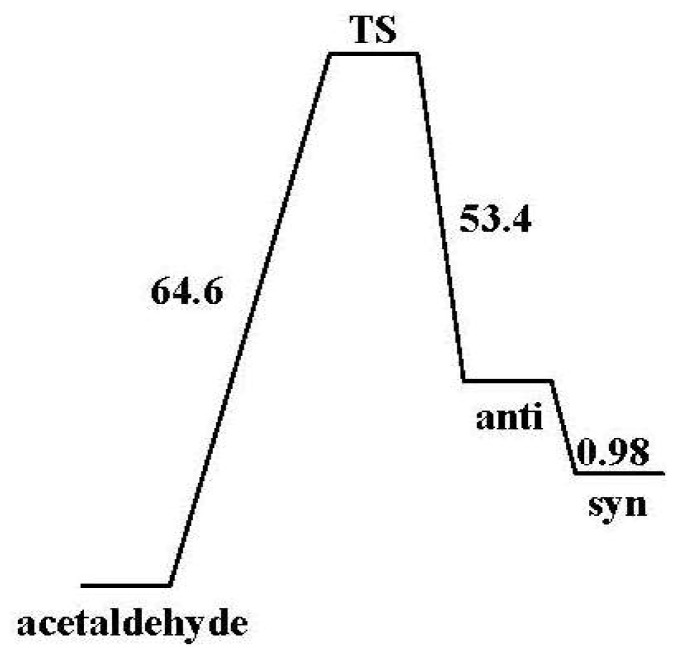
Activation Energies (kcal/mol) for the thermal tautomerization reaction of eclipsed acetaldehyde to produce syn and anti vinyl alcohol which were calculated using B3LYP/aug-cc-pvdz.

As shown in Table 4 and Figure 4 vinyl alcohol conformers lie 10.71 to 12.85 kcal/mol above the eclipsed acetaldehyde. These values are in line with the experimental [11] value of 9.80 ± 1.90 kcal/mol; and agree satisfactorily with those estimated by theory [12,15]. All our calculated syn/anti relative stabilities (0.98–1.16 kcal/mol) favored the syn form. They were in excellent agreement with the observed [23] range of 1.08 ± 0.14 kcal/mol. Thus, our best calculated activation energy (Figure 4) of 64.6 kcal/mol for the forward reaction and 54.38 kcal/mol for the reverse reaction is obtained by utilizing B3LYP/aug-cc-pvdz level of chemistry.

#### 2.2.3. Natural Bond Orbital (NBO) Analysis

**Figure 5:**
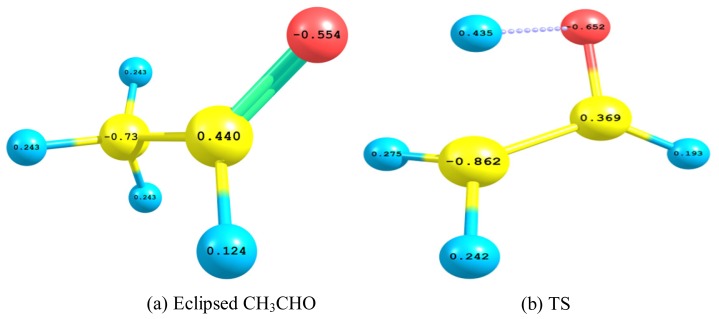
Natural atomic charges of eclipsed acetaldehyde and its TS which were calculated by using B3LYP/aug-cc-pvdz level of theory. (a) Eclipsed CH_3_CHO (b) TS

The natural atomic charges of eclipsed acetaldehyde (Figure 5a) and its TS (Figure 5b), which were calculated by using B3LYP/aug-cc-pvdz model chemistry, are depicted. For the eclipsed acetaldehyde, each of the three methyl hydrogen atoms acquires a positive charge of +0.243e. In contrast, the aldehyde hydrogen atom has a less positive charge of +0.193e. The methyl carbon is negatively charged (−0.73e) while the aldehyde carbon is positively charged (+0.44e) and the oxygen atom is negatively charged (−0.554e). In the TS the methyl hydrogen atom, in syn arrangement with the oxygen atom, developed more positive charge (+0.435e) and consequently partially bonded to the methyl carbon and oxygen atoms; which both gained extra negative charges of −0.862e and −0.652e respectively. This electron delocalization can be explained in terms of forming a four-membered ring TS, as a step forward for producing vinyl alcohol. This intuition is confirmed by earlier theoretical studies [11,12].

Table 5 lists the second order perturbation energies for eclipsed acetaldehyde and its TS which were calculated by using B3LYP/aug-cc-pvdz chemistry model. In eclipsed acetaldehyde there are very strong hyperconjugative interactions between the oxygen atom (LP) and the C–C bond (18.90 kcal/mol) and C2–H4 bond (23.59 kcal/mol). In comparison, there are also moderate hyperconjugation between the methyl C–H bonds and the C=O bond (σ_C1–H1_→σ*_CO_ and σ_C1–H3_→σ*_CO_) of 5.33 kcal/mol each; and between C1–H2 and C2–H4 bonds (σ_C1–H2_→σ*_C2–H4_ and σ_C2–H4_→σ*_C1–H2_ of 3.03 and 2.53 kcal/mol respectively). For the TS; the oxygen atom LP interaction with the C–C (3.56 kcal/mol) and C2–H4 (10.53 kcal/mol) were subdued, while the C1–H2 and C2–H4 interaction was enlarged (30.26 kcal/mol). This strong hyperconjugation led to the weakness of the C1–H2 bond in comparison to other bonds; as suggested by Guo and Goodman [25] when they investigated barrier forces in acetaldehyde. Thus this huge change in hyperconjugation has eased the migration of H2 from C1 to the oxygen atom. This conjecture is further supported by an emerging extremely strong delocalization interaction between the oxygen atom LP and C1–H2 bond (n_O2_→σ*_C1–H2_) of 96.08 kcal/mol. This latter interaction was nonexistent in the precursor; and thus supported the formation of a partial bonding between the oxygen and hydrogen atoms as a step forward for the formation of vinyl alcohol.

## 3. Computational Methods

All the computational methods have been performed using the Gaussian-09 suite [26]. The electronic energies at the optimized geometries of the eclipsed and bisected conformers of acetaldehyde and syn and anti vinyl alcohol, were obtained by using the perturbation method to second order (MP2) and the hybrid density functional theory (DFT) at the B3LYP method. Two basis sets, 6-311++G(d,p) and aug-cc-pvdz have been utilized.

The optimized geometries and electronic energies of the Transition State (TS) was requested with the QST2 keyword in the route section of the Gaussian program at MP2 and B3LYP methods and using 6-311++G(d,p) and aug-cc-pvdz basis-sets. A saddle point of first-order (one imaginary frequency) was located for the TS. Frequency calculations were then performed for the TS; as a prerequisite for running Intrinsic Reaction Coordinates (IRC) calculations. The normal modes of the imaginary frequencies and the IRCs were analyzed to reveal displacements in the bond lengths and angles involving the atoms of interest. GaussView Version 3.0 [27] program suite was used to visualize these displacements.

In order to obtain a complete description of the hyperconjugative interactions and Lewis-type orbital energies of the two conformers of acetaldehyde and the TS, the version 3.1 of the Natural Bond orbital (NBO) package [28] was used. DFT calculations have been carried out by the B3LYP method using the 6-311++G(d,p) and aug-cc-pvdz basis sets.

## 4. Conclusion

- The MP2 and DFT/B3LYP methods with the 6-311++G(d,p) and aug-cc-pvdz basis sets have been used to probe (mainly through NBO calculations): (a) the origin of relative stability preference for the eclipsed acetaldehyde over its bisected counterpart and (b) the elucidation of the 1,3-proton shift mechanism of the tautomerization reaction of eclipsed acetaldehyde and vinyl alcohol.
- The elected model chemistries addressed the problems investigated in a consistent and complementary manner. It is apparent that the relative stability of eclipsed/bisected acetaldehyde conformers is both basis set and method dependent. In comparison to the aug-cc-pvdz basis set, the 6-311++G(d,p) gave lower comparable values (1.02 and 1.09 kcal/mol) at both MP2 and B3LYP methods. Thus, we anticipate that a modern technology experimental reexamination of eclipsed/bisected acetaldehyde would lead to a difference in stability in the order of 1.06 ± 0.04 kcal/mol. As such it will be in line with our lower limits of 1.02 and 1.09 kcal/mol.
- The chemistry models have overestimated the dipole moments of the acetaldehyde conformers (eclipsed form always has a higher value) in comparison to the experimental [16] value of 2.750 ± 0.006 Debye. The MP2/aug-cc-pvdz level proved to be superior over other tested chemistry levels in reproducing the dipole moments of eclipsed and bisected acetaldehyde of 5.5% and 1.7% higher, respectively, compared to the experimental value [16].
- The Lewis 2-electron localized structure favored the bisected conformer of acetaldehyde over that of the eclipsed one. Apparently this originated from the minimal steric hindrance. However, the overall preference for the eclipsed conformer arose mainly from the hyperconjugative interactions. In particular, the vicinal antiperiplanar (σ_C1–H2_→σ*_C2–H4_ and σ_C2–H4_→σ*_C1–H2_) interactions played the major role in the competiveness of the eclipsed conformer.
- The DFT/B3LYP method produced lower barrier heights than those estimated by MP2 model. In contrast, the aug-cc-pvdz basis set achieved the lowest activation energies of 64.61 kcal/mol.
- The TS showed geometrical features that described the emergence of a four-membered ring species. This is achieved through an intramolecular migration of H2 accompanied by hybridization changes on heavy atoms. The NBO analysis of both eclipsed acetaldehyde and TS is in line with this intuition. The support for this prediction is based on the strong hyperconjugative interactions between the orbitals of the C1–H2 and C2–H4 bonds. This led to the weakness of the C1–H2 bond and a consequent intramolecular 1,3-proton shift between donor and acceptor sites.

## Figures and Tables

**Table 1:** The calculated electronic energies (a.u.), relative energies (kcal/mol) and dipole moment (D) of Eclipsed and Bisected Acetaldehyde obtained by using B3LYP and MP2 methods at 6-311++G(d,p) and aug-cc-pvdz basis sets.

	MP2/6-311++G(d,p)	MP2/aug-cc-pvdz	B3LYP/6-311++G(d,p)	B3LYP/aug-cc-pvdz
*E*_Full_ Eclipsed	−153.44965823	−153.41831870	−153.88225230	−153.85321084
*E*_Full_ Bisected	−153.44803394	−153.41641243	−153.88051576	−153.85128459
Δ*E*_T_/kcal/mol	1.02	1.19	1.09	1.21
*E*_L_ Eclipsed	-	-	−153.606588212	−153.587544947
*E*_L_ Bisected	-	-	−153.613900827	−153.595745153
Δ*E*_L_/kcal/mol			4.59	5.15
Delocalization Energy/kcal/mol			5.68	6.35
Energy Gain = Δ*E*_T_/kcal/mol			1.09	1.20
Exptl. a Δ*E*_T_		1.162 kcal/mol		
μ/D Eclipsed	2.943	2.907	3.376	3.360
μ/D Bisected	2.835	2.802	3.293	3.282
Exptl. b μ/D		2.750 ± 0.006 D		

a Experimental energy difference between eclipsed and bisected conformers of acetaldehyde taken from Reference [3];

b Experimental dipole moment of acetaldehyde taken from Reference [16].

**Table 2:** Second-order Perturbation Energy ((*E*_(2)_)/kcal/mol) estimates of the hyperconjugative energies of the Eclipsed and Bisected Conformers of Acetaldehyde calculated by using density functional theory (DFT)/B3LYP method at aug-cc-pvdz basis set.

Interaction	Eclipsed	Sum	Interaction	Bisected	Sum
σ_C1–H1_→σ^*^_CO_	5.33	20.26	σ_C1–H1_→σ^*^_CO_	4.51	17.56
σ_C1–H1_→π^*^_CO_	2.02		σ_C1–H2_→σ^*^_C2–H4_	1.34	
σ_C1–H3_→σ^*^_CO_	5.33		σ_C1–H2_→σ^*^_CO_	4.68	
σ_C1–H3_→π^*^_CO_	2.02		σ_C1–H3_→σ^*^_C2–H4_	1.34	
σ_C1–H2_→σ^*^_C2–H4_	3.03		σ_C1–H3_→σ^*^_CO_	4.69	
σ_C2–H4_→σ^*^_C1–H2_	2.53		-	-	

n_O1_→σ^*^_CC_	1.53	45.09	n_O1_→σ^*^_CC_	1.38	44.36
n_O1_→σ^*^_C2–H4_	1.07		n_O1_→σ^*^_C2–H4_	1.06	
n_O2_→σ^*^_CC_	18.90		n_O2_→σ^*^_CC_	19.13	
n_O2_→σ^*^_C2–H4_	23.59		n_O2_→σ^*^_C2–H4_	22.79	

Sum	65.35	65.35	Sum	61.92	61.92

**Table 3:** Optimized geometry a, bond lengths in Å and angles in degrees, of acetaldehyde, Transition State (TS) and syn and anti vinyl alcohol which were obtained by using B3LYP/aug-cc-pvdz level of theory.

Definition	CH_3_CHO	TS	Syn CH_2_CHOH	Anti CH_2_CHOH
C1–H1	1.102 (1.086) b	1.093	1.088 (1.070) c	1.087 (1.073) d
C1–H3	1.103 (1.086)	1.098	1.092(1.079)	1.089 (1.078)
C1–H2	1.096 (1.079)	1.507	-	-
C–C	1.504 (1.501)	1.415	1.337 (1.326)	1.335 (1.315)
C2–H4	1.118 (1.114)	1.099	1.090 (1.086)	1.093 (1.075)
C–O	1.212 (1.216)	1.285	1.366 (1.372)	1.372 (1.352)
OH	-	1.302	0.967 (0.969)	0.963 (0.941)
H1C1H3	109.57 (108.3)	113.78	117.89 (118.8)	118.85 (119.9)
H1C1C2	109.32 (109.2)	122.22	119.74 (119.5)	119.55 (119.9)
CCO	124.71 (123.9)	110.51	126.94 (126.2)	122.15 (122.7)
H4C2O	119.97 (117.5)	119.12	110.47 (110.7)	115.73 (115.7)
H1CCO	−121.85	−152.96	180.00	180.00
H3CCO	121.85	66.50	0.00	0.00
H2CCH4	−179.99	168.17	180.00	180.00
H2CCO	0.002	−9.09	180.00	180.00

a The optimized geometry obtained by this work. The values between brackets are the experimental geometries of:

b acetaldehyde taken from References [3,21];

c syn vinyl alcohol taken from Reference [22] and

d anti vinyl alcohol obtained from Reference [23].

**Table 4:** Zero-Point Corrected a Electronic Energies in a.u. and activation energies, relative stabilities (Δ*E*_1_ and Δ*E*_2_) b in kcal/mol of eclipsed acetaldehyde, Transition state (TS) and syn and anti vinyl alcohol, using B3LYP and MP2 methods at 6-311++G(d,p) and aug-cc-pvdz basis sets.

System	CH_3_CHO	TS	Δ*E*_1_	SYNCH_2_CHOH	Δ*E*_2_	ANTICH_2_CHOH
B3LYP/6-311++G(d,p)	−153.82889034	−153.72316138		−153.81118301		−153.80933272
Activation energy	66.35	-	11.70	55.23	1.16	54.07
B3LYP/aug-cc-pvdz	−153.7998880	−153.69692622		−153.78350726		−153.78194342
Activation energy	64.61	-	10.71	54.33	0.98	53.35
MP2/6-311++G(d,p)	−153.39641833	−153.28889802		−153.37687106		−153.37501719
Activation energy	67.47	-	12.85	55.20	1.16	54.04
MP2/aug-cc-pvdz	−153.36503858	−153.26034014		−153.34649926		−153.34470879
Activation energy	65.70	-	12.20	54.07	1.13	52.94

a Scaling factors taken from Reference [24];

b Δ*E*_1_ is the relative stability of acetaldehyde to vinyl alcohol and Δ*E*_2_ is the relative stability of syn and anti vinyl alcohols.

**Table 5:** Second-order Perturbation Energy ((*E*_(2)_)/kcal/mol) estimates of the hyperconjugative energies of the Eclipsed Acetaldehyde and its TS which were calculated by using DFT/B3LYP method at aug-cc-pvdz basis set.

Interaction/Compound	σ_C1H1_ →σ^*^_CO_	σ_C1H3_ →σ^*^_CO_	σ_C1H2_ →σ^*^_C2H4_	σ_C2H4_ →σ^*^_C1H2_	n_O2_ →σ^*^_CC_	n_O2_ →σ^*^_C2H4_	n_O2_ →σ^*^_C1H2_
CH_3_CHO	5.33	5.33	3.03	2.53	18.90	23.59	-
TS	9.50	2.37	30.26	2.53	3.56	10.53	96.08
